# Mitochondrial Kinases and the Role of Mitochondrial Protein Phosphorylation in Health and Disease

**DOI:** 10.3390/life11020082

**Published:** 2021-01-23

**Authors:** Veronika Kotrasová, Barbora Keresztesová, Gabriela Ondrovičová, Jacob A. Bauer, Henrieta Havalová, Vladimír Pevala, Eva Kutejová, Nina Kunová

**Affiliations:** 1 Institute of Molecular Biology, Slovak Academy of Sciences, Dúbravská Cesta 21, 845 51 Bratislava, Slovakia; veronika.kotrasova@savba.sk (V.K.); umbibarb@savba.sk (B.K.); gabriela.ondrovicova@savba.sk (G.O.); jacob.bauer@savba.sk (J.A.B.); umbiheha@savba.sk (H.H.); vladimir.pevala@savba.sk (V.P.); 2 First Faculty of Medicine, Institute of Biology and Medical Genetics, Charles University, 128 00 Prague, Czech Republic

**Keywords:** mitochondria, kinases, phosphorylation, disease

## Abstract

The major role of mitochondria is to provide cells with energy, but no less important are their roles in responding to various stress factors and the metabolic changes and pathological processes that might occur inside and outside the cells. The post-translational modification of proteins is a fast and efficient way for cells to adapt to ever changing conditions. Phosphorylation is a post-translational modification that signals these changes and propagates these signals throughout the whole cell, but it also changes the structure, function and interaction of individual proteins. In this review, we summarize the influence of kinases, the proteins responsible for phosphorylation, on mitochondrial biogenesis under various cellular conditions. We focus on their role in keeping mitochondria fully functional in healthy cells and also on the changes in mitochondrial structure and function that occur in pathological processes arising from the phosphorylation of mitochondrial proteins.

## 1. Introduction

Cells continuously undergo numerous changes as a result of metabolic conditions, stress and pathological situations. Mitochondria are organelles that contribute substantially to managing all of these challenges. Generally, cells respond to varying conditions in two ways. One way is by synthesizing newly demanded proteins and degrading those whose level must be reduced. For mitochondria, a large amount of energy and careful signaling is needed to manage all the messages continuously flowing into and out of the organelle. The second way is to modulate protein functionality through post-translational modifications (PTMs), which enables a rapid adaptation by precise modulation of stability, structure and function of particular proteins. Proteins can be phosphorylated, succinylated, ubiquitinated, sumoylated, acetylated, glycosylated, nitrated and malonylated. PTM is a reversible process which allows modified proteins to be quickly returned to their original state when conditions are altered. Phosphorylation is a PTM that is involved both in signaling and in modifying protein functions and is frequently employed in mitochondria. Accordingly, proteomic studies have found that ~40% of the organellar proteome is phosphorylated [1,2,3]. Moreover, to date, at least 25 protein kinases have been reported to either associate with mitochondria or have mitochondrial substrates (for rev. see [4,5,6,7]). Both Ser/Thr kinases, such as AKT [8,9], CK2 [10,11], GSK-3β [12], PKA [10,11], isoforms of PKC [13,14], PINK1 [15,16,17], components of the MAPK signaling pathway (JKN, p38) [18,19], and LRRK2 [20], and tyrosine kinases, such as ABL [21,22] and members of the SRC family (FYN [23], SRC [24,25,26], LYN [27], FGR [28,29], CSK [30] and EGFR [31]) have been identified to function within mitochondria. The phosphorylation of mitochondrial proteins has been connected with the dysregulation of mitochondrial functions that is found in neurodegenerative diseases, cancer, diabetes, heart dysfunction and aging. Here, we provide an overview of mitochondrial kinases, their substrates and functions and the diseases associated with their dysregulation.

## 2. Kinases on the Outer Mitochondrial Membrane

Protein kinases selectively modify other proteins by covalently attaching a phosphate group to a specific amino-acid residue, thus allowing cells to rapidly and reversibly adapt to changing cellular conditions. The human kinome consists of more than 500 kinases [32], which potentially affect nearly every cellular process, including those taking place in mitochondria. A growing number of mitochondrial proteins have been found to be phosphorylated, but only a fraction of the known protein kinases has been found either within the organelle itself or associated with its outer (OMM) or inner mitochondrial membranes (IMM) [4,5,33].

Almost no protein kinase has a known mitochondrial targeting signal, so their transport into mitochondria is still poorly understood. Several studies have shown that some kinases originally present in the cytosol use proteins embedded in the OMM as a scaffold [34,35]. For example, the cytosolic Jun N-terminal kinase (JNK) interacts with the membrane-bound protein Sab (SH3-binding protein 5), and disrupting this interaction has a neuroprotective effect in rat Parkinson’s disease (PD) models and cerebral ischemia [36,37]. Interestingly, an anchoring protein for one kinase might be a substrate for another. In this case, Sab was shown to be phosphorylated by p38 (a mitogen-activated protein kinase, MAPK) [38]. A second example is the cAMP-dependent protein kinase A (PKA), which can be docked to the OMM by the A-kinase anchoring proteins (AKAPs), for example AKAP1 or the AKAP84 found in sperm [34,39]. AKAPs usually bind specifically to PKA regulatory subunit II, although several dual specificity AKAPs, which bind both regulatory subunits I and II, have been reported [40]. AKAP1 was shown to be phosphorylated by AMPK (5′-AMP-activated protein kinase) [41]. AMPK, in addition to AKAP1, has only two other currently known mitochondrial substrates, ACC2 (acetyl-CoA carboxylase 2), a lipid metabolism protein, and the mitochondrial fission factor (MFF), the receptor of mitochondrial fission [42,43].

PKA is also associated with other mitochondrial compartments, though the process by which PKA moves across the OMM into the matrix is still not fully understood. PKA-mediated phosphorylation affects a wide variety of mitochondrial functions, including protein import, oxidative phosphorylation, steroid hormone metabolism, mtDNA maintenance and apoptosis (see below for these). It also plays a role in mitophagy, a selective mitochondrial degradation process. PKA regulates mitophagy by modifying DRP1 (dynamin-related protein 1), a protein belonging to the dynamin family of large GTPases. Phosphorylation of DRP1 at Ser637 inhibits mitophagy and promotes mitochondrial fusion, most likely by increasing its binding to mitochondrial elongation factor 1/2 (MIEF1/2) [44,45]. A decrease in mitochondrial fission is also caused by the phosphorylation of DRP1 by AMPK, which plays a protective role in nervous system mitochondria, as well as by DRP1 phosphorylation by the Ca^2+^/calmodulin-dependent protein kinase (CAMK) that acts in cardiomyocytes [46,47]. Mitochondrial fission is promoted, on the other hand, by DRP1 phosphorylation at Ser585 by CDK5 (cyclin-dependent kinase 5) [48], by modification of Tyr266 by the Abelson tyrosine kinase (ABL) [22], and by phosphorylation at the highly conserved Ser616 by ERK1/2 (extracellular receptor kinase 1/2), which increased mitochondrial fragmentation in human pancreatic adenocarcinoma [49]. In an Alzheimer’s disease (AD) mouse model, GSK-3β (glycogen synthase kinase 3β) causes phosphorylation of DRP1 at Ser40 and Ser44, which promotes its GTPase activity, thereby increasing mitochondrial division and neuronal apoptosis [50]. In dopaminergic mouse neuronal cells (SN4741), DRP1 Ser616 is phosphorylated by p38 MAPK, which increases its mitochondrial translocation, resulting in mitochondrial dysfunction and neuronal loss [51]. The phosphorylation of DRP1 associated with mitophagy inhibition is abolished by the activity of *PTEN*-induced putative kinase 1 (PINK1) [52]. PINK1, when targeted into the mitochondria, disrupts the anchoring of PKA to the OMM by inhibiting its binding to AKAP1 and thus ensuring the necessary fission of damaged mitochondria for organellar degradation [53].

PINK1 is one of the few mitochondrial kinases to contain a true mitochondrial targeting sequence (others are creatine kinase [54] and PAK5 (p21-activated kinase 5) [55]). In healthy mitochondria, PINK1 translocates into the matrix and, after phosphorylating its substrates, immediately undergoes rapid cleavage by the mitochondrial proteases MPP (mitochondrial processing peptidase) and PARL (presenilins-associated rhomboid-like protein) with further targeting to the proteasome [56,57,58,59]. When mitochondria lose their membrane potential, as a result of aging or chemical exposure, auto-phosphorylated PINK1 accumulates in the OMM, forming a complex with proteins of the TOM (translocase of the outer membrane) machinery [60,61]. The kinase domain of PINK1 also phosphorylates the E3-ubiquitin ligase Parkin, which activates its enzymatic functions and triggers the processes leading to mitophagy [62].

Mutations in genes encoding either PINK1 or Parkin are implicated in the pathogenesis connected with Parkinson’s disease [63,64,65,66] along with mutations to LRRK2 (leucine-rich repeat kinase 2), which is also found in the OMM [67,68]. Mutated LRRK2 confers aberrant kinase activity, which results in the increased phosphorylation of its substrates, leading to malfunctions in various mitochondrial processes including increased reactive-oxygen species (ROS) production [69], mitochondrial dynamics [70], and mitophagy [71] and also dysregulated calcium homeostasis [72].

Two other proteins involved in the mitochondrial fusion/fission cycle are also protein kinase targets. Mitofusin 1 (Mfn1) when phosphorylated by ERK1/2 inhibits mitochondrial fusion [73] while mitofusin 2 (Mfn2) when phosphorylated by JNK becomes a substrate for the proteasome and promotes the fragmentation of mitochondria in human osteosarcoma cells [74]. Phosphorylation of Mfn2 by PKA at Ser442 inhibits the Ras signaling pathway in rat vascular smooth muscle (VSM) cells, suppressing VSM cell growth in vascular disorders such as post-angioplasty restenosis and atherosclerosis [75].

Additionally, two less well-known protein kinases have also been found associated with the OMM. Mammalian target of rapamycin (mTOR) is an important regulator of cell growth, protein and lipid synthesis, autophagy and other mitochondrial functions (for rev. see [76]); it was found in the outer membrane of human T-cell mitochondria [77,78]. A direct substrate for this membrane-associated protein is still unknown, however it has been proposed to have a role in apoptosis and leukemia [79]. At least in three cancer cell lines, mTOR phosphorylates hexokinase II, which catalyzes one of the first steps of glucose metabolism in mitochondria. This phosphorylation may have a decisive role in switching between glycolytic pathway and oxidative phosphorylation, thus increasing tumor cell resistance [80,81]. In neural cells, including those in neuroblastoma, PAK5 was shown to be localized to the OMM [55,82] where it phosphorylates the pro-apoptotic protein Bad, thereby inhibiting apoptosis [55,82].

To date, protein kinases confirmed to be localized to the OMM are Ser/Thr kinases. In addition to PKA [83,84], JNK [85], ERK1/2 [86], p38 MAPK [87], PINK1 [88], CDK5 [48], LRRK2 [89], mTOR [77], PAK5 [82], CAMK [47] and AMPK [90], they also include cyclin-dependent kinase 11 (CDK11) [91], casein kinases 1 and 2 (CK1, CK2) [92,93], and three isoforms of protein kinase C (PKC α, PKC δ and PKC ε) [94,95,96]. All these kinases phosphorylate diverse mitochondrial substrates, resulting in many different alterations to organellar dynamics, protein import, metabolism, respiratory complex activity and apoptosis, all of which will be more extensively discussed in the following chapters.

## 3. Phosphorylation in Mitochondrial Import Machinery

Mitochondria are semiautonomous organelles with their own transcription-translation system. Although they are able to synthesize some of their functionally important components, most of the essential ions, small molecules, RNAs [97,98] and proteins [99] need to be imported from the cytoplasm.

**Figure 1:**
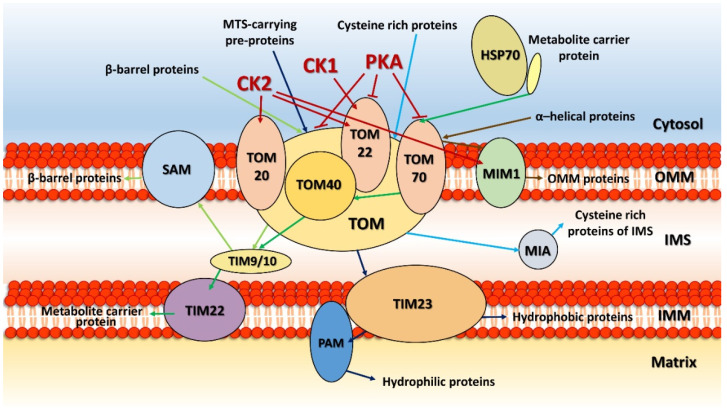
The major mitochondrial protein import pathways and the influence of protein kinases in *Saccharomyces cerevisiae*. MTS (mitochondrial targeting sequence)-carrying pre-proteins are imported through the TOM and TIM23 complexes. Proteins containing a hydrophobic sorting signal are embedded into the IMM while hydrophilic proteins are sent into the mitochondrial matrix through the PAM (protein import motor) complex. Cysteine-rich proteins are imported by the TOM and MIA protein translocation systems. The precursors of β-barrel proteins are translocated through the TOM and TIM9/10 complexes and are sorted and assembled by the SAM complex. The precursors of metabolite carriers are imported through TOM, TIM9/10 and TIM22, and several α-helical OMM proteins are imported by the MIM complex. Protein kinase A (PKA) phosphorylates the TOM40 and TOM22 precursors in the cytosol, thereby impairing their translocation and assembly into the mature TOM complex. PKA also phosphorylates TOM70 which abolishes its interaction with the metabolite carrier/chaperone HSP70 complex. CK1 phosphorylates TOM22, promoting its assembly, whereas CK2 phosphorylates both, TOM20 and TOM22. Phosphorylation of TOM22 facilitates its interaction with TOM20 and stimulates its assembly into the mature TOM complex. CK2 further phosphorylates MIM1 enhancing its stability and promoting the MIM1-dependent import of the TOM20 and TOM70 precursors. OMM, mitochondrial outer membrane; IMM, mitochondrial inner membrane.; IMS, intermembrane space.

Indeed, most mitochondrial proteins are synthesized in the cytosol and only afterwards transported to their designated location within the organelle. Protein kinases alter these processes in two different ways: they either phosphorylate the mitochondrial signaling pre-sequences of these proteins [100] or they modify the components of the import machinery [99,101]. These components include the outer-membrane complexes translocase of the outer membrane (TOM), mitochondrial import complex (MIM) and sorting and assembly machinery (SAM) and the inter- and inner-membrane systems translocase of the inner membrane (TIM) and mitochondrial intermembrane space assembly (MIA) (Figure 1). Moreover, the outer-membrane TOM complex was shown to directly cooperate with both MIA and TIM in the inner mitochondrial membrane, as well as the SAM complex through the activity of the inter-membrane proteins TIM9 and TIM10 [102].

The phosphorylation of mitochondrial pre-sequences was studied in the plant *Arabidopsis thaliana* [100]. By searching the PhosPhAt 4.0 Database [103], 103 mitochondrial pre-proteins with one or more experimentally determined phosphorylation sites were found. These included subunits of respiratory complexes I–V and proteins with functions in the tricarboxylic acid (TCA) cycle, protein translocation and degradation, RNA editing, transport and several others. More detailed studies of the respiratory complex subunits CAL2, SDH1-2, COX5b1, ATP17, F1β-1, F1β-2, and F1β-3 have shown that the N-terminal pre-sequences could be phosphorylated by several kinases with different but overlapping specificities, which might influence the protein import into plant mitochondria [100].

The effect of phosphorylation on the *Saccharomyces cerevisiae* mitochondrial import complexes has been well described [10,11,104,105] (Figure 1). A mass spectrometry analysis of purified *S. cerevisiae* mitochondria allowed the different phosphorylation sites of proteins in the TOM and MIM complexes to be mapped [105]. Several serine and threonine residues were found to be phosphorylated, including Ser84, Ser87, Thr92, Ser168 and Ser172 in TOM20; Thr5, Ser20, Ser44, Ser46, Thr76, and Thr129 in TOM22; Ser2,Thr5, Ser11, Ser50, Ser54, Ser77, and Thr220 in TOM40; Ser10 in TOM5; Ser16 in TOM6; Ser6 in TOM7; Ser65, Thr66, Ser69, Ser78, Ser166, Ser174, Thr228, Thr234, Thr232, and Thr520 in TOM70; Ser55, Ser73, and Ser96 in TOM 71; and Ser12 and Ser16 in MIM1 (all from [106]).

TOM20, TOM22 and TOM70 function as receptors for the import of nuclear-encoded precursor proteins into the mitochondria. TOM20 and TOM70 are responsible for the initial recognition of mitochondrial precursors that are later transferred to the central receptor, TOM22, and, through the action of TOM40, transported across the outer mitochondrial membrane. TOM20 is mainly phosphorylated by casein kinase 2 at Ser172, and inactivation of CK2 was shown to reduce the activity of both TOM20 and TOM22 [105]. Interestingly, however, the amino-acid substitution mutants TOM20^S172A^ (no phosphorylation at Ser172) and TOM20^S172E^ (mimicking phosphorylation at Ser172) showed no abnormalities in mitochondrial protein composition, TOM assembly or protein import [105,107]. In addition to being a CK2 substrate, TOM22 is also phosphorylated by casein kinase 1 [107]. CK2 modifies TOM22 at two proximal serines, Ser44 and Ser46, both of which are present in the active TOM complex and are already phosphorylated in the cytosol. On the other hand, the CK1-mediated phosphorylation of Thr57, which is required for the assembly of the TOM complex, takes place only after it has been imported into the mitochondria. Phosphorylation and TOM22 assembly are significantly increased in mitochondria obtained from yeast grown on glucose (a fermentable carbon source) compared to those from cells grown on glycerol (non-fermentable). Further observation showed that phosphorylations to TOM22 mediated by CK1 and CK2 are important for the recruitment and assembly of TOM20 into the TOM complex, thereby emphasizing the complex effects of phosphorylation on the formation and stability of the OMM import machinery and the biogenesis and dynamics of mitochondria. This is even more apparent for the phosphorylation of TOM22, TOM70 and TOM40 by PKA. PKA phosphorylates TOM22 at Thr76 in the cytosol, which decreases its binding to mitochondria and thus considerably reduces its membrane integration. Both CK1 and PKA, therefore, have opposites effects on TOM22 under fermentable growth conditions. Whereas CK1-mediated phosphorylation stimulates TOM22 function, PKA strongly inhibits it; thus, CK1 moderates the negative effect of PKA. In cells grown on a fermentable carbon source, TOM70 is phosphorylated at Ser174 in a PKA-dependent manner [105] and the PKA treatment of mitochondria isolated from such cells showed that only a fraction of TOM70 is phosphorylated in vivo. Here, it seems that phosphorylation does not affect either the targeting or the oligomerization of TOM70 but does influence its protein-transport function. TOM70 is the main receptor of non-cleavable hydrophobic protein carriers (like AAC, the phosphate carrier and the dicarboxylate carrier) [108,109], which are delivered to TOM70 by the cytosolic HSP70 complex. TOM70 phosphorylation at Ser174 substantially reduces the interaction between TOM70 and the precursor carrier protein complex with Hsp70, thereby leading to a great impairment in the import of metabolic carriers into the mitochondria [11,105].

TOM40, a hydrophobic channel that enables the majority of mitochondrial proteins to be imported [110], is phosphorylated by PKA at Ser54 in the cytosol [104]. This phosphorylation blocks the formation of the SAM–TOM40 complex intermediate important for the assembly of TOM40 into the mature TOM complex and inhibits its import into the OMM. The whole process is negatively regulated by the presence of a fermentable carbon source like glucose or sucrose when PKA is rapidly activated [111]. Under these conditions, the assembly of a functional TOM complex is impaired, leading to lower mitochondrial activity. When switched to nonfermentable growth conditions, PKA activity lowers, and TOM40 is imported into the OMM in response, forming the channel that allows the effective protein transport and increased mitochondrial activity [104].

MIM1 is located in the OMM [112,113]. Although it is not a structural part of the TOM complex, it is required for the membrane insertion of the TOM complex components, including TOM20, TOM70 and TOM40. MIM1 is phosphorylated at Ser12 and Ser16 by CK2, partially in the cytosol and fully after its transport into the mitochondria [105], which strongly influences the assembly of TOM70 and TOM20 into the functional TOM complex.

Several MS analyses have shown that TOM complex components of human mitochondria are also phosphorylated [114,115,116,117,118], although more detailed functional studies are still lacking. Phosphorylated serine, threonine and tyrosine residues were found in TOM20 (Ser55, Ser135, Ser138), TOM22 (Ser15, Thr43, Thr45, Thr125), TOM40 (Tyr129, Ser142, Thr144, Ser157, Tyr194, Thr254, Thr255, Thr273, Ser317, Ser320, Thr338) and TOM70 (Ser19, Thr33, Thr85, Ser91, Ser96, Ser110, Tyr153, Tyr156, Ser253, Tyr260, Ser279, Tyr310, Tyr327, Tyr339, Ser434, Tyr601, Tyr607) [106].

Presently, it can only be assumed that some of these modifications may play roles in human diseases, especially cancer as, to the best of our knowledge, no individual studies have been done to date. In humans as in yeast, glucose repression substantially inhibits mitochondrial protein import and thereby reduces mitochondrial biogenesis. In summary, phosphorylation of the import machinery components regulates the translocation of proteins at multiple levels and allows for very precise cellular responses to metabolic and energetic demands.

## 4. Kinases and Metabolism

Mitochondria are the centers of energy production for most eukaryotic organisms. They integrate the metabolism of carbohydrates and amino acids and fatty acids to generate energy in the form of ATP. The key enzyme is the mitochondrial pyruvate dehydrogenase complex (PDC), which catalyzes the oxidative decarboxylation of pyruvate to acetyl-CoA [119]. This irreversible conversion links the glycolysis occurring in the cytosol to the Krebs cycle (citric or tricarboxylic acid (TCA) cycle) in the mitochondria and the synthesis of fatty acids and steroids [120,121]. PDC is one of the largest and most complex multi-enzyme systems known [122]; its activity is controlled by multiple reversible phosphorylations. In fact, PDC was one of the first phosphorylation-regulated enzymes to be described [123].

The mammalian PDC is composed of four major components: a thiamine diphosphate-dependent pyruvate dehydrogenase (E1), a dihydrolipoamide transacetylase (E2) containing covalently bound lipoyl groups, the flavoenzyme lipoamide dehydrogenase (E3), and an E3-binding protein (E3BP) [120]. It also contains a family of four pyruvate dehydrogenase kinases (PDKs 1–4), which inactive it through phosphorylation, and two pyruvate dehydrogenase phosphatases (PDPs 1 and 2), which dephosphorylates the enzyme to its active form [121]. The PDK isoenzymes belong to the ATPase/kinase superfamily [124,125,126] and phosphorylate three serine residues in the E1 α subunit: Ser293 (phosphorylation site 1), Ser300 (phosphorylation site 2) and Ser232 (phosphorylation site 3) [120,121,127,128]. Phosphorylation of Ser264 completely inactivates PDC, while phosphorylation of sites 2 and 3 maintains a low catalytic activity [129]. All four PDKs modify site 1 and 2; site 3 is phosphorylated only by PDK1 [129,130]. The activity of the PDKs has to be tightly regulated. Short-term regulation is carried out by metabolites; acetyl-CoA and NADH (both are formed by the pyruvate dehydrogenase (PDH) reaction and fatty acid β-oxidation), activate the complex while the pyruvate generated by glycolysis or circulating lactate inhibit it [131].

The PDC can also be modulated by cytosolic JNK, one of the MAPKs [119]. Phosphorylation-activated JNK was found in the OMM of primary cortical neurons where it was translocated in a response to H_2_O_2_ or anisomycin treatment (an activator of JNK/p38) and the PDC was shown to be its specific phosphorylation target [119]. Phosphorylated PDC reduces its pyruvate dehydrogenase activity, causing the cell to shift to anaerobic pyruvate metabolism, which is characterized by increased levels of lactate in the cytosol [119]. In aging rat heart, the amount of the phosphorylated, and therefore inactive, form of PDH was significantly increased, however the PDK activity was only slightly lower than that in younger rat cardiomyocytes [132]. The JNK-mediated inhibition of PDH and concomitant reduction of energy production seems to be especially important in tissues with high energy uptake like heart and brain or during brain aging [133]. Deficiencies in PDH regulation caused by malfunctions of JNK were shown in the pathogenesis of neurodegenerative disorders including Parkinson’s and Alzheimer’s diseases [134,135].

Hypoxia-induced glycolysis in the cytosol is coupled to suppressed mitochondrial pyruvate metabolism and oxidative phosphorylation [136]. Thus, pyruvate accumulated in the cytosol is converted to lactate (the so-called Warburg effect) [137] and the subsequent metabolic changes associated with acidosis promote cell proliferation and tumorigenesis [136,138,139]. Hypoxic conditions induce a signaling pathway leading to the translocation of cytosolic PGK1 (phosphoglycerate kinase 1), the first ATP-generating enzyme in the glycolytic pathway [140], into the mitochondria [136]. Mitochondrial PGK1, whose expression is upregulated in breast [141], pancreatic [142], ovarian [143], metastatic gastric [144] and colon cancer [145] and hepatocellular carcinomas [146], phosphorylates, and thereby activates, PDK1 at Thr338 [136]. The enhanced activity of PDK1 results in the inactivation of PDH by phosphorylation of Ser293, leading to the suppression of pyruvate utilization and ROS production in mitochondria, which is a poor prognosis for patients with glioblastoma [136].

PDC was further shown to be phosphorylated by the Ser/Thr kinase TPKI/GSK-3β (Tau protein kinase I/glycogen synthase kinase 3β) [147], which was originally identified as a tau-kinase associated with brain microtubules, possibly involved in phosphorylation of the Tau protein that occurs in Alzheimer’s disease [148]; this kinase is presently known to play a key role in the molecular pathophysiology of many neurodegenerative diseases [12]. TPKI/GSK-3β translocates into the mitochondria upon stress-induction, where it phosphorylates and inhibits PDH in rat hippocampal cell cultures, again resulting in dysfunctional mitochondria and neuronal death [12,147].

A phosphoproteomic study of mitochondria isolated from potato tubers confirmed that the PDC is massively phosphorylated. The phosphorylation of formate dehydrogenase (FDH, which catalyzes the oxidation of formate to CO_2_, reducing NAD^+^ to NADH in the process) at Thr76 and Thr333 was also observed [149]. Phosphorylation of FDH decreased in response to higher levels of pyruvate, formate and NAD^+^, whereas its activity was strongly increased by low mitochondrial oxygen concentrations, suggesting that phosphorylated FDH has a role in hypoxia [149].

In addition to the pyruvate and formate dehydrogenases, several other mitochondrial metabolic enzymes are phosphorylated by kinases. Aconitase, the enzyme catalyzing the second step of the TCA cycle (and also responsible for the metabolic regulation of iron and the stabilization of mtDNA) [150,151], is modified by the FGR tyrosine kinase at Tyr71, Tyr544 and Tyr665 in rat brain mitochondria [29,152]. All three tyrosines reside in a region that is highly conserved in all eukaryotes, which indicates that these phosphorylations are likely important for the stability and enzymatic activity of the protein [29]. FGR phosphorylates succinate dehydrogenase complex subunit A (SDHA) at Tyr535 and Tyr596 [29,152]. In *S. cerevisiae*, phosphorylation of citrate synthase, which mediates the first step of TCA cycle, at Ser462 completely abolishes its catalytic activity by inhibiting homodimer formation [153]. A mass spectrometry analysis of *Solanum tuberosum* mitochondria found that NAD-isocitrate dehydrogenase, succinyl-CoA ligase subunits α/β, NAD-malate dehydrogenase and 2 isoforms of NAD-malic enzyme were phosphorylated [149] (the physiological significance of these modifications remains largely unknown) [154].

Steroid hormone biosynthesis in mitochondria is also controlled through modifications mediated by protein kinases. The first and rate-limiting step in steroidogenesis is the transfer of cholesterol from the OMM to the IMM, which is facilitated by the Steroidogenic Acute Regulatory protein (StAR) [155]. Its inactive form resides within the IMM or matrix. Upon hormone stimulation, StAR is translocated to the OMM in a mitochondria-fusion-dependent manner and activated [156]. Here, phosphorylation mediated by PKA followed by ERK1/2 occurs, both of which are necessary for maximal steroid production [155,157]. StAR contains two consensus PKA-phosphorylation sites, Ser55/56/57 and Ser194/195, which are conserved across all eukaryotes [158,159]. PKA phosphorylation allows the binding of a cholesterol molecule to the hydrophobic pocket of StAR [157]. ERK1/2 phosphorylates StAR at Ser232, which elevates its activity and increases cholesterol transport [155,157].

The StAR activity cycle enables many cholesterol molecules to be metabolized per unit of StAR protein. When unnecessary, StAR may be dephosphorylated, and thereby deactivated, by mitochondrial phosphatases, but left intact to become available later to bind another cholesterol molecule or it may be translocated into the mitochondrial matrix and degraded by the ATP-dependent protease Lon [160].

## 5. OXPHOS System Modification

**Figure 2:**
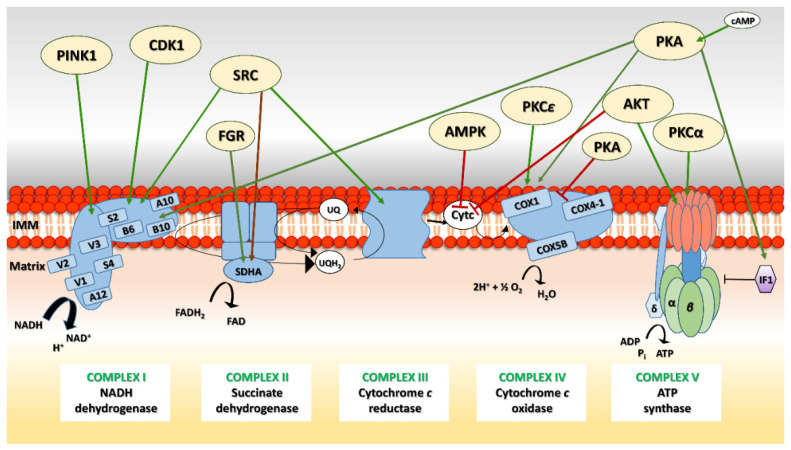
The phosphorylation of mammalian OXPHOS system components. The OXPHOS system consists of five protein complexes (NADH dehydrogenase, succinate dehydrogenase, cytochrome c reductase, cytochrome c oxidase and ATP synthase) embedded in the IMM. The protein kinase-regulated subunits are highlighted as follows: stimulation in green, repression in red and no effect in brown. Phosphorylation by PKA, PINK1, CDK1 and SRC increases the activity of complex I (NADH dehydrogenase). Succinate dehydrogenase (complex II) is regulated by the action of the FGR and SRC kinases. The activity of cytochrome c (Cytc) oxidase (complex III) is upregulated by phosphorylation by the SRC kinase. The activity of cytochrome c is impaired upon phosphorylation by AKT and AMPK. Complex IV (cytochrome c reductase) is negatively regulated by PKA (independently of cAMP levels) and positively by the cAMP-dependent action of PKA and PKC ε. Phosphorylation of ATP synthase (complex V) is upregulated by AKT and PKCα. Phosphorylation of the inhibition factor IF1 by PKA abolishes IF1 binding and its inhibition of ATP synthase activity. IMM, inner mitochondrial membrane.

NADH and FADH_2_, the electron carrier molecules produced by the TCA cycle, transfer their electrons to oxygen in several steps through respiratory chain complexes embedded in the inner mitochondrial membrane. This metabolic pathway, called oxidative phosphorylation (OXPHOS), uses a series of enzymes to transport electrons and generate the proton gradient used in ATP production. The OXPHOS system consists of five protein complexes (NADH dehydrogenase, succinate dehydrogenase, cytochrome *c* reductase, cytochrome *c* oxidase and ATP synthase) each of which is made up of numerous subunits, some of which are encoded by the mitochondrial DNA, and regulated by various PTMs including phosphorylation [161] (Figure 2).

NADH dehydrogenase (complex I), the largest of the respiratory chain complexes, is conserved in all eukaryotes except several yeast species (e.g., *S. cerevisiae, Schizosaccharomyces pombe*) [162]. It catalyzes the oxidation of NADH by reducing coenzyme Q, and this process is associated with the translocation of protons across the IMM. NADH dehydrogenase is an L-shaped protein complex consisting of several cofactors and as many as 45 subunits, 14 of which form the evolutionarily widely conserved core of the enzyme [163,164]. A bioinformatics analysis identified several potential phosphorylation sites, indicating that phosphorylation is prevalent in various subunits of complex I and may play a role in its assembly and regulation [165].

In mouse fibroblasts, a nuclear-encoded 18 kDa subunit (referred to as AQDQ or the NDUFS4 subunit) was shown to become phosphorylated in elevated levels of intramitochondrial cAMP, leading to a twofold increase of complex I activity. The mouse, bovine, human and *Neurospora crassa* sequences of this protein have consensus phosphorylation sites in both the leader sequence and the mature protein [166], and are phosphorylated by the cAMP-dependent PKA [167,168]. The importance of the PKA recognition site within the NDUFS4 subunit was shown by studies on patients with a fatal neurological Leigh-like syndrome. An inherited autosomal recessive mutation of a 5 bp duplication in the *NDUFS4* gene results in a 14 amino-acid extension of the mature NDUFS4 form which causes the loss of the phosphorylation site required to activate complex I without affecting the assembly nor the activity of other complexes (i.e., cytochrome *c* oxidase) [169,170]. This decreased activity of complex I resulted in the increased production of ROS [171,172]. In addition to regulating complex I activity, PKA also phosphorylates a serine residue in the C-terminal part of NDUFS4 that is necessary for its translocation from the cytosol into the mitochondria [173].

In humans, the complex I subunit NDUFA10 is phosphorylated at Ser250 by PINK1 [174]. Studies in fly, mouse and human models demonstrated that *PINK1* mutations affect the activity of complex I and overall mitochondrial homeostasis by causing an impaired mitochondrial membrane potential, increased sensitivity to apoptotic stress and decreased complex I activity [174,175]. Mutations in PINK1 that prevent it from phosphorylating its substrates is one of the most significant causes of a recessive form of Parkinson’s disease (PD) [174].

Other complex I subunits are phosphorylated by the cyclin B1/CDK1 kinase [176]. In mouse and human cells, subunits NDUFV1, NDUFV3, NDUFS2, NDUFB6 and NDUFA12 have been observed to be phosphorylated; the phosphorylated proteins increase mitochondrial respiration and energy production, which is greatly needed in the later stages of the cell cycle [176]. Furthermore, complex I subunits, NDUFV2 and NDUFB10, were shown to be phosphorylated by the SRC tyrosine kinase [25,26]. Phosphorylation of both subunits increased the levels of respiration, electron transfer and cellular ATP content [25,26]. In humans, inhibiting SRC leads to a decrease in mitochondrial respiration [26], while higher levels of SRC were observed in various types of tumors, indicating that the SRC-mediated maintenance of mitochondrial functions may play a role in tumor cell proliferation [25].

Succinate dehydrogenase (SDH; complex II) is an enzyme with important dual roles in the respiratory chain and TCA cycle. The SDHA flavoprotein is phosphorylated at Tyr215 by c-SRC in vitro and in vivo [26]. Phosphorylation of SDHA has no effect on its respiratory activity but is important for efficient electron transfer through electron transfer chain (ETC) complex II and preventing ROS generation [26]. In male mice with defective SDHA Tyr215 phosphorylation, a low humoral immune response was observed with the suppression of both Ig production and formation of a germinal center against antigens [177]. SDHA is also regulated by the FGR kinase-mediated phosphorylation of Tyr604, which is highly conserved among mammals [28,29]. The absence or inhibition of respiratory complex I (e.g., by rotenone) leads to increased SDHA phosphorylation and a concomitant increase in complex II activity, which may be an efficient compensatory mechanism for electron transfer and FADH_2_ processing [28].

Not much is known about the phosphorylation of respiratory complex III. Several subunits were among the phosphoproteins identified by proteomic studies in human cells [178], although the functional significance of individual phosphorylations have not yet been elucidated. Here again, the SRC tyrosine kinase seems to be responsible for generally upregulating complex III activity. The same was shown for complexes I and IV, but not for the SRC-mediated decrease in activity of respiratory complex V [179].

Cytochrome *c* (Cytc) is a small protein containing a covalently bound heme group that functions as an electron carrier between mitochondrial ETC complexes III and IV. Mammalian Cytc contains several highly tissue-specific phosphorylation sites. Phosphorylated Tyr97, Tyr48, and Thr28 were found in heart, liver and kidney, respectively [180,181,182]. In rat renal Cytc, AMPK is responsible for the phosphorylation of Thr28, which lies close to the heme group and reduces its ability to transfer electrons to complex IV [180]. A similar effect was observed after introducing phosphorylation at Tyr48 [183]. Additionally, a phosphorylated Thr58 conserved in mammalian somatic Cytc isoforms (replaced with isoleucine in testis) was found in kidney and a site-specific Ser47 phosphorylated by AKT kinase (protein kinase B) in brain [182,184]. All these Cytc phosphorylations reduce the level of mitochondrial respiration, lower ROS generation, lower the mitochondrial membrane potential and decrease apoptotic activity (see Section 8 below) [180,182,183,184].

Human respiratory complex IV is composed of 13 subunits, three of which are encoded by mitochondrial DNA [185]. Various studies suggest that the phosphorylation of different subunits may lead to both inhibition and activation of complex IV under various pathophysiological conditions. Tyr304 in cytochrome *c* oxidase catalytic subunit I (COX1) is the target of a cAMP-dependent phosphorylation by an as yet unidentified tyrosine kinase [186]. Tumor necrosis factor-α (TNFα), a pro-inflammatory cytokine participating in the immune response in mammals, seems to be responsible for triggering the signal transduction leading to the activation of an unknown mitochondrial kinase modifying Tyr304 [187]. Like the Cytc phosphorylation mentioned above, phosphorylation of COX1 also leads to decreases in complex IV activity, ATP production and mitochondrial membrane potential [186,187].

Respiratory complex IV is also regulated by PKA, which has a regulatory role in hypoxia and ischemia. Elevated levels of PKA under hypoxic conditions or myocardial ischemia cause the hyperphosphorylation of several COX subunits, including COX1, COX4-1 and COX5b, which is associated with decreased complex IV activity [188]. Hypoxia-activated PKA, independently of mitochondrial cAMP levels, phosphorylates the COX5b subunit. Accordingly, the substitution of phosphorylated COX5b with a phosphorylation-resistant protein retained the activity of complex IV. Thereby, hypoxia-activated PKA responds to increased ROS production, induces PKA substrates phosphorylation and mediates signal for complex IV inhibition [189]. Under hypoxic and ischemic conditions, the levels of COX1, COX4-1 and COX5b are reduced [188]. Recent studies have shown that the mitochondrial protease Lon, which regulates the levels of mitochondrial nucleoid-associated proteins [190], also degrades COX subunits [191]. Lon, induced by hypoxia and ischemia, has a direct role in degrading phosphorylated COX4-1 and COX5b, whereas non-phosphorylated forms of these subunits were relatively resistant to Lon proteolysis [191]. In contrast, Acin-Perez et al. [192] described a PKA-mediated phosphorylation of COX4-1 on Ser58 that increased COX activity, probably by altering its ADP/ATP binding [192]. Other studies found that COX is regulated by protein kinase C, a Ser/Thr kinase PKC-***ε*** [193,194]. Activated PKC is translocated to various subcellular sites depending on cellular conditions. In mitochondria, hypoxia-induced PKC-***ε*** interacts with and phosphorylates COX4, which may have a protective role against hypoxia by activating complex IV [193,194].

ATP synthase (complex V) is the last enzyme in the OXPHOS system. It uses the energy stored in the proton gradient across the IMM created by the ETC to drive the synthesis of ATP. In potato tuber mitochondria, two phosphorylated complex V proteins have been identified, the δ′-subunit of the F1-ATPase and the β-subunit of the ATPase [195]. In human skeletal muscle, seven potential phosphorylation sites were found in the β-subunit of F1-ATP synthase [196]. Interestingly, patients with type 2 diabetes had downregulated levels of the phosphorylated F1-ATP synthase β-subunit [196]. These results suggested that this phosphorylation may be an important mechanism for regulating electron-coupled ATP synthesis, and that disturbances in it may contribute to the pathogenesis of insulin resistance and type 2 diabetes [197]. Insulin resistance occurs when cells do not respond well to insulin and, at the same time, cannot use glucose from blood for energy. Insulin also stimulates the translocation of AKT to mitochondria, whose activation increases complex V activity [198]. In patients with insulin-resistant type 2 diabetes, a decrease in the mitochondrial translocation of AKT and a related decrease in the activity of ATP synthase were observed [198].

The activity of ATP synthase is also regulated by inhibitory factor 1 (IF1) [199,200]. The binding of IF1 depends on its phosphorylation status. Dephosphorylated IF1, which is abundantly present in human carcinomas, inhibits both the synthase and hydrolase activities of complex V and upregulates aerobic glycolysis. A PKA-mediated phosphorylation at Ser39 abolishes IF1 binding to ATP synthase [199,201]. Further studies revealed that the PKCα-dependent phosphorylation of the α, β and δ subunits of ATP synthase leads to an increase in the activity of ATP synthase, which suggests that PKCα might have a role in the preservation of mitochondrial functions after cell injury [95,202].

ATP synthase subunits were also among those proteins identified by phosphoproteomic studies of human cancers, however the exact function of these phosphorylations is currently not known [118].

Uncoupled respiration is a specific respiratory process that occurs in the mitochondria of brown and beige adipose cells, also known as fat cells [203]. Brown adipose tissue (BAT) is involved in thermogenesis, when energy is dissipated into heat in response to conditions like hibernation, cold stress or food intake [204,205]. Mitochondrial dysfunction in adipocytes can lead to metabolic diseases, including type II diabetes and obesity [204]. Mitochondrial uncoupling protein 1 (UCP1) plays a major role in the uncoupled respiration in brown and activated beige adipocytes. UCP1 transfers protons from the intermembrane space back to the mitochondrial matrix before ATP synthase can use them, thereby producing heat rather than ATP [206,207]. In BAT, UCP1 is inhibited by free purine nucleotides: ATP, ADP, GTP, and GDP [208]. Upon induction of thermogenesis, specific hormones (e.g., norepinephrine) trigger a reaction cascade leading to cAMP production and activation of lipolysis through a PKA-mediated phosphorylation. Subsequently, PKA-phosphorylated enzymes break down lipid droplets into smaller free fatty acids, which in turn activate UCP1 [206,209].

Phosphorylation was also shown to regulate the transcription of the *UCP1* gene in several ways [210]. A phosphorylation reaction cascade involving the cAMP-dependent PKA and the p38 mitogen-activated protein kinase (p38 MAPK) leads to activation of transcription factor 2 (ATF-2), which increases the expression of *UCP1* [211]. PKA also directly upregulates *UCP1* transcription through the cAMP-response element binding protein (CREB) [206,210]. Shinoda et al. [212] also found that the formation of brown and beige adipocytes is suppressed by casein kinase 2 (CK2) and CK2 inhibition stimulates the UCP1-mediated thermogenesis [212]. Yet another study showed that the phosphorylation of UCP1 at Ser51 increases upon cold stress. It is thought that phosphorylation increases the activity of UCP1, though the responsible kinase remains unknown [213].

## 6. Mitochondrial Quality Control

Mitochondria are highly dynamic cellular structures that have developed their own systems for detecting dysfunction and eliminating the damage. These include quality control mechanisms consisting of a sophisticated network of mitochondrial chaperones and proteases, which are together responsible for stabilizing, folding, refolding or completely removing proteins. When the mitochondrial proteome suffers extreme damage, mitophagy or cell death can occur [214,215,216]. The main components of the mitochondrial protein quality control (MPQC) system include the heat-shock proteins Hsp60, Hsp70 (mortalin), Hsp90 (TRAP1), the Lon protease [217] and the caseinolytic peptidase ClpXP (absent in *S. cerevisiae* and *S. pombe*). Phosphorylation plays an important role in regulating the functions of all of them, and their inhibition or aberrant activation often leads to pathological conditions.

Several chaperones in plant and mammalian mitochondria are phosphorylated. For example, the small heat-shock protein (sHSP) Hsp22 in *Zea mays* was found to be phosphorylated in vivo [218]. In vegetative tissues, Hsp22 forms large oligomers of 9 or more subunits and is constitutively expressed at very low levels but is strongly induced in response to heat stress and protects mitochondrial respiratory complex I [219,220,221]. In mammalian systems, the phosphorylation of similar heat shock proteins (e.g., HSP27) is mediated by the activity of the mitogen-activated protein kinase MAPK [222,223], and usually shifts the distribution of sHSPs towards smaller species [224], however, the precise effect of phosphorylation on plant sHSPs remains elusive. Additional proteomic analyses revealed phosphorylations of mitochondrial Hsp70 (*Pisum sativum*), Hsp90 and chaperonin Hsp60 (*Solanum tuberosum*) [149]. Moreover, a tyrosine in Hsp75, a member of the HSP70 family, was found to be phosphorylated in rat hepatoma cells treated with peroxovanadate (a non-specific inhibitor of tyrosine phosphatases) [225].

The human mitochondrial chaperone TRAP1 (TNF receptor-associated protein 1), a member of the highly conserved HSP90 family, is an important heat-shock protein which is involved in regulating mitochondrial energy metabolism in both healthy and damaged cells [226,227,228]. TRAP1 was found to be constitutively phosphorylated at low levels and phosphorylation significantly increased in response to oxidative stress [229]. Under these conditions, the Ser/Thr kinase PINK1 acts as a major phosphorylation agent. The level of TRAP1 phosphorylation is directly linked to the cytoprotective function of PINK1, which counteracts oxidative-stress induced apoptosis by suppressing the release of cytochrome *c* from the mitochondria [229]. In Parkinson’s disease (PD), mutations in PINK1 abolish its ability to phosphorylate TRAP1, thereby increasing Cytc release into the cytosol and triggering the cell death pathway; this might at least partially explain the mitochondrial dysfunction leading to neurodegeneration in PD patients [229].

TRAP1 expression is strongly induced in several types of tumors [230], where it is frequently correlated with progression, metastasis and disease recurrence [226,231]. In carcinogenesis, TRAP1 may function as an effector of the deregulated extracellular signal-regulated protein kinase 1/2 (ERK1/2) signaling pathway [232]. ERK1/2 directly phosphorylates TRAP1 at two site-specific serines, Ser511 and Ser568, which promotes its activation and the concomitant downregulation of mitochondrial respiratory complex II (succinate dehydrogenase) [232]. The resulting accumulation of intracellular succinate causes pseudohypoxia by stabilizing the hypoxia-inducible transcription factor (HIF-1), thereby activating the HIF-1 responsive genes independently of cellular oxygen levels, leading to further metabolic changes and progression to malignancy [227,233].

Interestingly, TRAP1 is also one of the proteins phosphorylated during sperm capacitation in humans, a process which is correlated with an increase in the tyrosine phosphorylation of several proteins by PKA [234]. These include two PKA-membrane anchoring proteins, AKAP3 and AKAP4, and VCP/p97, a member of the AAA family (ATPases associated with diverse cellular activities) that presumably links sperm capacitation and acrosome reaction [234].

Mitochondrial Hsp60, together with its co-chaperone Hsp10, typically serves to maintain mitochondrial homeostasis by assisting in the folding and trafficking of other proteins [235,236,237]. It also has other roles in mtDNA transactions and apoptosis, and even more roles occur upon its translocation into the cytosol or extracellular space (for rev. see [238]). The phosphorylation of Hsp60 is involved in both physiological and pathological processes: phosphorylation of Tyr227 and Tyr243 were reported in non-small lung cancer cell lines as well as in mouse hyperglycemic myoblasts [239,240]. The SRC-mediated phosphorylation of Tyr227 was also observed during rotavirus infection and resulted in Hsp60 degradation, thereby delaying the import of viral non-structural proteins (NSP4) into the mitochondria. This delay prevents premature host cell apoptosis, thus giving the rotavirus enough time to replicate [241]. A tyrosine-phosphorylated Hsp60 was also found in the surface activation of sperm cell capacitation mentioned above [242].

The mitochondrial ATP-dependent protease Lon is one of the main components of the MPQC system and is involved in the degradation of the damaged, misfolded or unfolded proteins that accumulate in the mitochondrial matrix [216,243]. Lon has been shown to be subjected to at least three different PTMs, including phosphorylation [106,244,245,246]. Lon expression is induced by oxidative stress, endoplasmic reticulum (ER) stress, proteotoxic stress, starvation or hypoxia [247]. Lon upregulation has been observed in ischemic rabbit hearts and macrophages under hypoxic conditions [248]. In hypoxia associated with cancer, Lon upregulation was linked to the stabilization of HIF-1 and its binding to the *LON* promoter [249,250]. In prostate adenocarcinoma, the upregulation of Lon was connected to the action of the AKT Ser/Thr kinase [245]. AKT phosphorylates Lon at two serines, Ser173 and Ser181, in the N-terminal domain, which increases its proteolytic activity. This enhanced protein degradation in turn restores the activity of ETC complexes II and V, and increases the oxygen consumption rate, ATP production and cell migration [245]. Conversely, plant mitochondrial Lon was found to be phosphorylated at Ser654 in response to a *Xanthomonas citri* infection. This phosphorylation rapidly impairs its proteolytic activity [251], preventing it from degrading HrpG, a transcription regulator of the *hrp/hrc* genes. Since these genes are not expressed, they cannot act against the *X. citri* virulence resulting in necrosis in citrus fruits and green leaves [252].

Yet another mitochondrial protease involved in MPQC is the hetero-oligomeric protein complex, ClpXP, which mediates a proteolytic removal of misfolded proteins [253,254] that are often induced in response to stress [255,256,257]. A functional Clp protease is formed from two parts, a heptameric peptidase ClpP and a hexameric ATP-dependent chaperone ClpX, which combine to create a higher-order structure that resembles the barrel of the cytoplasmic 26S proteasome [258,259]. ClpP lacks ATPase activity and therefore retains only a low level of peptidase activity when ClpX is missing [260] while ClpX, as a regulatory ATPase, can still perform its chaperone function even in the absence of ClpP [261]. The mutual interaction of both ClpX and ClpP subunits is needed to activate the proteolytic function, in which ClpX unfolds a protein substrate using an ATP-driven translocation through its central pore into the ClpP proteolytic chamber [262].

ClpP has a well-characterized role in the mitochondrial unfolded protein response (mtUPR) of eukaryotes. This is a retrograde signaling pathway that maintains mitochondrial protein homeostasis in response to mitochondrial-specific stress [263]. In mammals, the mtUPR might also be induced by changing the stoichiometry of the proteins encoded by mitochondrial and nuclear genomes. This is correlated with the upregulation of ClpP and Hsp60, which together minimize mitochondrial protein aggregation [255,264]. On the other hand, reduced levels of ClpP alters mitochondrial and cellular morphology, reduces cell proliferation, and results in lower mitochondrial respiration, lower oxygen consumption rate and increased ROS production [263]. MtUPR was shown to be crucial for tumor cell survival during anti-cancer treatment [265], and is dependent on the activation of the cytosolic double-stranded-RNA-activated protein kinase PKR [266]. PKR or protein kinase R is a Ser/Thr kinase constitutively and ubiquitously expressed in vertebrate cells that was first identified as a protector against viral infections [267,268]. In mice lacking a functional PKR, no mtUPR-responsive proteins (e.g., chaperonin 60) were activated upon stress induction [266].

ClpXP was also shown to be dramatically upregulated in primary and disseminated human tumors, which often correlates with reduced patient survival [269]. In these cases, an interaction between ClpXP and TRAP1 were observed with survivin, an inhibitor of apoptosis, which acts as a scaffold bringing ClpP and TRAP1 together [269]. Seo et al. [269] found that succinate dehydrogenase subunit B (SDHB) of respiratory complex II was a key substrate whose loss profoundly affected mitochondrial bioenergetics and lead to decreased oxygen consumption, loss of ATP production and mtUPR activation (no other ETC complex was affected) [265]. Thus, ClpXP, TRAP1 and survivin provide a mechanism for ensuring mitochondrial homeostasis in tumors, making any of these proteins a potential target for anti-cancer therapy.

Proteomic analyses identified phosphorylated residues in both ClpP and ClpX. ClpX was found to be phosphorylated at several serines, threonines and tyrosines, some of which were directly in the ATPase domain. The most frequently phosphorylated residue was Ser617, which was identified by more than 20 phosphoproteomic studies, predominantly in different types of lung cancer tissues [106]. For the proteolytic part of the Clp protease, phosphorylations were found at Ser169, Ser173, Ser181, Ser231, Thr189 and Thr277, all of which, except Thr277, were found in the ClpP protease domain [106]. To date, however, no further experimental data are available on how phosphorylation influences the ClpXP proteolytic or chaperone activities.

## 7. Nucleoid and Ribosomes (mtDNA Maintenance, Transcription and Protein Synthesis)

Although a large number of mitochondrial proteins are known to be phosphorylated, only a few examples are currently known from those involved in mtDNA maintenance and transactions. Even there several protein kinases could under various, often pathological conditions, translocate into the mitochondria and influence the mtDNA-associated processes.

Mitochondrial DNA is compacted into nucleoids, nucleoprotein complexes that are associated with the IMM [270]. The most abundant group of nucleoid proteins are the mtDNA-packaging proteins which contain one or two high-mobility group (HMG)-box domains that are critically involved in mtDNA binding and compacting (for review see [271]). The most studied and best characterized mitochondrial HMG-box proteins are the *S. cerevisiae* Abf2 (ARS-binding factor 2) and the human mitochondrial transcription factor A (TFAM), both of which are known to be phosphorylated [272,273,274]. Indeed, nuclear HMG-box proteins are frequently phosphorylated in response to changes in physiological state. In the nucleus, the Ca^2+^-phospholipid-dependent protein kinase C (PKC) seems to be responsible for many of the phosphorylation events, and has been identified as the modifier of several chromatin-binding HMG-box proteins including mammalian HMG1/2 [275,276], insect HMG1 [277], human HMG14/17 [278] and both human and murine HMGY/I isoforms (these are also phosphorylated by the protein kinases Cdc2 and MAPK [279,280]). In all these proteins, phosphorylation has a negative effect on their DNA-binding properties, significantly weakening even abolishing their affinity for various DNA substrates [277,278,279].

Yeast mitochondrial Abf2 can be modified by PKA, which rapidly and specifically phosphorylates Thr21 and Ser22 in the N-terminal segment of the Abf2 HMG-box 1 in vitro [272]. Like its nuclear counterparts, phosphorylation in this region inhibits its DNA-binding affinity for various types of DNA substrates and the DNA super-coiling activity of Abf2 itself. As the *ABF2* gene seems to be constitutively expressed, it is tempting to speculate that the inhibition of its activities might be the means by which mtDNA transactions are regulated in yeast. Supporting this hypothesis is the fact that the amount of Abf2 protein in mitochondria changes more as a consequence of fluctuations in mtDNA copy number rather than in changes in *ABF2* gene expression [281]. Despite this, such a challenging hypothesis still requires much more thorough investigation.

Like Abf2, TFAM, the mammalian mtDNA-packaging protein, possesses several potential phosphorylation sites in its two HMG boxes. For example, in vitro PKA-mediated phosphorylation affects several serines, with Ser55, Ser56 and Ser61 in HMG-box 1 being the most notable [273]. In the crystal structure of the TFAM–LSP (light-strand promoter) DNA complex [282], these residues were in direct contact with the DNA and could thus play a decisive role in mtDNA-binding regulation. Indeed, phosphorylation in HMG1 impaired the ability of TFAM to bind DNA and to activate the transcription of mitochondrial DNA [273]. In the mitochondrial transcription complex, TFAM acts as an auxiliary transcription factor in recruiting the mitochondrial RNA polymerase (POLRMT) and mitochondrial transcription factor B (TFB2M) to the mtDNA promoter sites [283]. The mass spectrometry analysis of Wang et al. [274] identified Ser177 in HMG2 as the ERK1/2 phosphorylation site. Elevated ERK kinase activity has been shown in the degenerating substantia nigra neurons of patients with PD and Lewy body disease (LBD), however it is still unclear whether activated ERK localizes to the mitochondria normally or only gains entry following mitochondrial injury [86]. Nonetheless, its presence within the mitochondria is sufficient to induce mitophagy [284]. In cancer, ERK-dependent signalling enhances aerobic glycolysis, a metabolic pathway preferred by highly proliferating cells because it provides intermediates compatible with rapid growth [285]. Mimicking the ERK-mediated TFAM phosphorylation on Ser177 again negatively affects its DNA-binding ability, especially the light-strand promoter sequence, with consequent effects on the levels of mtDNA transcription, mitochondrial respiration and respiratory chain subunit expression. This may reveal an additional mechanism by which ERK1/2 downregulates mitochondrial functions [274].

In *Arabidopsis thaliana* chloroplasts, MFP1 (MAR-binding filament-like protein 1) serves as the major DNA-binding protein and has functions comparable to those of mitochondrial Abf2 or TFAM [286]. MFP1 was shown to associate with the thylakoid membranes and bind plastid DNA in a non-specific manner [286]. Its DNA-binding activity is largely inhibited after the phosphorylation of either of two adjacent serine residues, Ser604 or Ser605, by serine protein kinase CK2, whose α subunit homolog, cpCK2α, is a component of the chloroplast transcription apparatus [287]. Three of the 10 currently known nucleoid-associated proteins in plastids [288] have a DNA-binding activity that can be influenced by phosphorylation. In addition to MFP1, sulfate reductase (SiR), a bifunctional enzyme acting in DNA compaction and sulfur assimilation [289,290] and SWIB-4, a domain of SWI/SNF complex B functioning in cpDNA packaging [291] were both shown to lose their DNA-binding abilities when phosphorylated [291,292].

Several proteins involved in mtDNA transcription and replication have either been found in a phosphorylated form or have been shown to be substrates of a particular protein kinase. In *S. cerevisiae*, a PKA-mediated phosphorylation has been reported for MTF1 (mitochondrial transcription factor 1) [293], a protein that interacts with the mtRNA polymerase and facilitates promoter-specific transcription initiation [294]. MTF1, evolutionarily related to bacterial rRNA methyltransferases, is a functional homolog of human TFB2M [295,296] and binds DNA in a non-sequence-specific manner. Two independent MS analyses [297,298] found a single phosphorylation on Ser93, however direct information on its consequences for mtDNA promoter binding or mitochondrial transcription initiation remain unknown.

Additionally, the activity of mTERF1 (mitochondrial termination factor 1), a factor mediating mtDNA transcription termination in mammalian mitochondria, was shown to be modulated by several phosphorylations and only the phosphorylated protein allowed accurate transcription ending to occur [299]. Interestingly, dephosphorylation of mTERF1 abolishes its termination activity, but not its DNA-binding ability [299], showing that these two activities are separable and the binding of the protein to DNA is necessary but not sufficient to terminate transcription [300].

Mammalian CREB (cAMP-response element binding protein) is also activated by phosphorylation. CREB normally resides in the nucleus, where it acts as a transcription factor in the signaling pathways involved in synaptic transmission and neuronal survival [248]. Despite having no mitochondrial localization signal, phospho-activated CREB was found in the mitochondria of adult rat brain [301]. Following PKA-mediated activation on Ser133, CREB is able to enter mitochondria (through the TOM complex, with the help of mtHSP70) and bind CRE-like sequences in the non-coding region of the mtDNA [302,303]. Its depletion in mitochondria decreases the expression of mitochondrially encoded genes, resulting in the down-regulation of mitochondrial respiration which is one of the characteristics of the mitochondrial dysfunction associated with neurodegenerative disorders [302].

In the maturation of mitochondrial mRNA transcripts prior to translation in *S. cerevisiae*, an as yet unidentified protein kinase phosphorylates the 55-kDa RNA-binding protein DBP (dodecamer-binding protein) [304]. This phosphorylation activates DBP’s high-affinity binding to conserved dodecamer sequences in the 3′ UTR region of mature mitochondrial mRNAs [305]. This region also serves as a target for the mitochondrial degradosome (mtEXO), the major regulator of yeast mitochondrial RNA turnover [306,307,308]. Interestingly, the precise role of DBP in *S. cerevisiae* mtRNA processing has not been described, although its unique DNA-binding activity is known to be regulated by a reversible phosphorylation [305].

In humans, the mitochondrial translational elongation factor (EF-Tu) is phosphorylated in response to ischemia in rabbit cardiomyocytes [309]. EF-Tu is a GTPase that binds aminoacyl-tRNAs and brings them to the ribosome. In bacteria, phosphorylation of Thr382 of the EF-Tu homolog prevents such ternary complex formation and protein translation is largely inhibited [310]. Similarly, mitochondrial EF-Tu from isolated ischemic hearts had multiple phosphorylation sites, including the same highly conserved threonine, and at least one additional serine residue. The cytosolic Ser/Thr kinase JNK, which translocates into mitochondria upon ischemic heart injury [311], was suggested to be a possible candidate. However, follow-up experiments excluded both JNK and the p38 MAP kinase because no change occurred in the levels of EF-Tu phosphorylation upon their immuno-depletion [309]; the kinase responsible for EF-Tu modification in mitochondria still remains to be identified.

The proper maintenance and expression of the mitochondrial genome in all organisms depends on the faithful copying of mtDNA. The mitochondrial single-stranded DNA-binding protein (mtSSB) is a key component of the mitochondrial replisome [312], and is fundamentally involved in keeping the human mtDNA copy number levels constant. It undergoes protein kinase-mediated changes, though these changes are often related to pathological processes. mtSSB was shown to be phosphorylated by SRC, a mitochondrially localized non-receptor tyrosine kinase. SRC is an oncogene which has high expression or activity in several types of solid tumors, including glioblastoma [313], prostate [314], breast [315], pancreas [316], colon [317], and lung cancers [318], and it is associated with increased invasiveness, metastatic potential and lower patient survival [319]. The presence of SRC in the mitochondria was confirmed in breast cancer, prostate carcinoma and bone osteosarcoma cell lines [25,320], and was correlated with altered mitochondrial respiration [321]. In highly metastatic breast cancer cells, mtSSB was shown to be downregulated [322] and similar downregulation was observed when mtSSB was phosphorylated [24]. Phosphorylation of mtSSB on Tyr73 decreases mtDNA replication activity, leading to a reduction in mtDNA levels [24]. This reduced amount of mtDNA may further lower the expression of mitochondrially-encoded proteins (including the respiratory complex subunits), followed by OXPHOS deficiency and more aggressive phenotypes in breast cancer tumors [24].

## 8. Mitochondrial Kinases in Apoptosis

**Figure 3:**
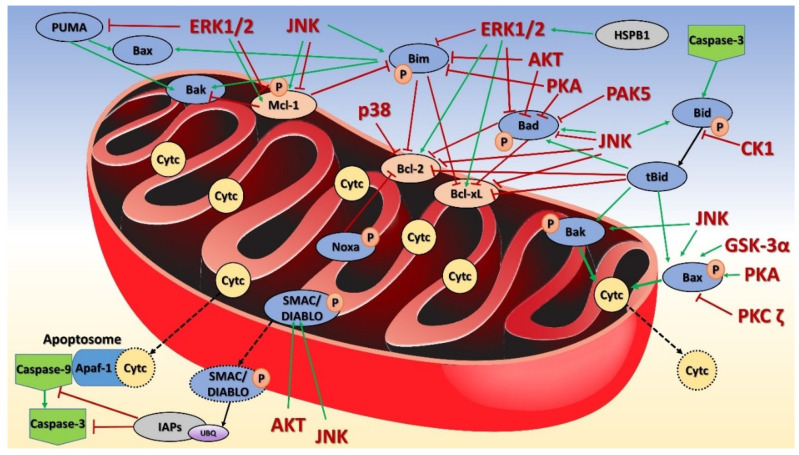
The apoptotic pathways influenced by mitochondrial protein phosphorylation. Pro-apoptotic (circled in blue) and anti-apoptotic proteins (circled in light red) are phosphorylated by the action of various protein kinases (shown in red) as described in Section 8. The dashed black arrows represent a translocation of the indicated protein out of the mitochondrion. Cytc, cytochrome *c*; P, phosphate group; UBQ, ubiquitin.

The role of mitochondria has been univocally established in apoptosis, a major form of regulated cell death that affects the processes of development, differentiation and tissue homeostasis. The central regulatory role is played by the Bcl-2 protein family that are divided into pro-apoptotic (Bax, Bak, Bok, Bad, Bid, Bik, Bim, Bmf, Noxa, Puma) and anti-apoptotic (Bcl-2, Bcl-xL, Bcl-W, Mcl-1) groups. Their dysregulation is often implicated in a wide variety of diseases, including excessively apoptotic atrophy or the insufficiently controlled cell proliferation of cancer. The activities of the Bcl-2 family and other apoptosis-related proteins must therefore be strictly regulated either on the transcriptional or the post-translational levels, which involves the phosphorylation and dephosphorylation activities of many mitochondrial kinases and phosphatases (Figure 3 for mitochondrial kinases).

The mitochondrial pathway of cell death requires mitochondrial outer membrane permeabilization (MOMP) to release soluble proteins from the mitochondrial intermembrane space, especially Cytc, which activates initiator caspase 9, which cleaves and activates the executioner downstream caspases [323]. The other proteins released include Smac/DIABLO and OMI, which block the caspase inhibitor XIAP (X-linked inhibitor-of-apoptosis protein), thereby enabling apoptosis. In healthy cells, Bax localizes to the cytosol and Bak to the mitochondria, and both are inactive and able to shuttle between those compartments [324,325,326]. Bax and Bak can be directly activated by binding a subclass of BH3-only proteins (Bid, PUMA and Bim) [327]. Upon activation, Bax accumulates in the mitochondria. The phosphorylation of Bid at Ser64 and Ser66 in mouse mitochondria (Ser65 and Ser67 in humans) by CK1 prevented its activation and subsequent binding to Bax and Bak, thereby inhibiting Fas-mediated apoptosis and the release of Cytc from mitochondria [328,329]. Phosphorylation of Bid at Tyr54 is frequently found in phosphoproteomic studies of leukemic, breast and gastric cancer tissues, though its precise role has not yet been described [106].

In early apoptosis, Cytc functions as a peroxidase of cardiolipin, a mitochondria-specific lipid that binds to Cytc, in the presence of H_2_O_2_ [330,331]. This pro-apoptotic event promotes the dissociation of Cytc from the IMM and its release into the cytosol [332]. Of the five Cytc phosphorylation sites noted above (Section 5), only Tyr48 seems to influence apoptosis. Phosphorylation of Tyr97 showed only a small reduction in caspase-9 activity in vitro [333,334], and a phosphomimetic substitution of Tyr48 reduced caspase 3 activity by ~70% but left the cardiolipin peroxidase activity of Cytc unchanged [183].

As mentioned before, the MAPKs (JNK, ERK1/2, and p38) and PKA exhibit transient mitochondrial localization in response to various cellular stimuli and stresses which alter the regulation of cell death pathways. Their main targets are the members of the Bcl-2 protein family, which are largely phosphorylated [335]. In cerebral ischemia, JNK1 may be an early mitochondrial effector responsible for JNK-mediated apoptosis [4,37]. The numerous kinases and phosphatases localized to the OMM coordinate organellar and cellular physiology by modulating the roles of the Bcl-2 protein family in metabolism, autophagy, and apoptosis. The interplay between ROS and the members of the Bcl-2 family can determine whether a cell undergoes apoptosis [336]. Bcl-2 “senses” the extent of ROS production through the phosphorylation of its Ser70 by JNK, which releases it from mitochondria, and thereby alters ETC function, mitochondrial autophagy and antioxidant capacity [337].

In humans, the pro-apoptotic protein Bad is phosphorylated at four serines, Ser74, Ser75, Ser99 and Ser118. Ser75 seems to be phosphorylated by ERK1/2, which has been correlated with several human cancers [106,115,338,339,340]. Likewise, Ser99 is a major AKT phosphorylation site [341]. In mice, the JNK kinase phosphorylates Bad at Ser128, which subsequently mediates its oligomerization and promotes its pro-apoptotic effect in primary neurons by antagonizing the ability of growth factors to inhibit Bad-mediated apoptosis [342,343]. On the other hand, JNK reduces apoptosis in interleukin-3-mediated cell survival by phosphorylating Bad at Thr201 and inactivating it [344]. Moreover, in mice, PAK5 can phosphorylate Bad on Ser112 and thereby prevent its migration to mitochondria, which, consequently, increases the levels of the anti-apoptotic protein Bcl-2 and inhibits apoptosis [82]. Mitochondrial PKA and AKT also phosphorylate mouse Bad at Ser112 and Ser155 (Ser75 and Ser118 in humans), as well as Bim, another pro-apoptotic protein, at Ser83 [345] and Ser65 during trophic factor deprivation (TFD) [346,347], both of which reduce their inhibitory effects on the anti-apoptotic proteins Bcl-2 and Bcl-xL, thereby promoting cell survival [348,349]. Bim was also found to be phosphorylated by the action of ERK1/2. Here, the interaction of ERK1/2 with heat shock protein B1 (HSPB1) facilitates the degradation of phosphorylated Bim. This is disturbed in Charcot-Marie-Tooth disease, where HSPB1 mutations lead to high levels of Bim and the cells exhibit increased susceptibility to ER stress-induced cell death [350].

The human pro-apoptotic pore-forming protein Bax is phosphorylated at several threonine (Thr22, Thr86, Thr135, Thr140, Thr167 and Thr174) and serine residues (Ser60, Ser87, Ser163 and Ser184) [106], the physiological effects of most of which remain unclear. The PKA-mediated phosphorylation of Bax at Ser60 is known to promote its mitochondrial translocation and MOMP [351], and the phosphorylation of Ser163 by GSK-3β was observed in the mitochondria of human embryonic kidney cells and cerebellar granule neurons [352]. The phosphorylation of Thr167, located between the last two α-helices, by JNK promotes Bax oligomerization and induces apoptosis [353]. In both small cell and non-small cell lung cancers, however, Bax is phosphorylated at Ser184, presumably by the PKC ζ isoform. Ser184 is inside the last α-helix, which is responsible for mitochondrial membrane localization, and this phosphorylation led to a retention of the protein in the cytoplasm and inhibition of apoptosis [354].

In healthy cells, Bak, the second mitochondrial pore-forming pro-apoptotic protein, is also phosphorylated at several sites, including Tyr108, Tyr110, Ser117 and Thr148 [106]. Dephosphorylation of Tyr108 and Ser117 seems to be an important regulatory step, where dephosphorylated Tyr108 initiates the cell death process by conformational changes in the OMM [355]. Ser117 located in the hydrophobic groove of Bak is involved in binding to other pro-apoptotic partners (BH3-only proteins). Its dephosphorylation mediates further multimerization and, consequently, pore formation, which enables Bak pro-apoptotic function [356].

Bcl-xL is a critical anti-apoptotic protein that plays a role in normal embryogenesis, human erythropoiesis and promotes the survival of differentiating pancreatic cells [357,358,359]. Apoptosis is promoted when Bcl-xL residues Thr47 and Thr115 are phosphorylated in human myeloid leukemia cells by stress-activated JNK [360]. Outside its function in apoptosis, Bcl-xL was shown to be phosphorylated at Ser49 and Ser62 during normal cell cycle progression and checkpoints [361,362]. The PLK3 (Polo-like kinase 3) phosphorylated Bcl-xL at Ser49 accumulates in the centrosomes during G2 checkpoint induced by DNA damage and during the final stages of mitosis and cytokinesis. Here, phosphorylated Bcl-xL translocates to the microtubule-associated dynein motor protein and prevents chromosome instability [361]. Whereas Bcl-xL phosphorylated at Ser62 by PLK1 and MAPK14/SAPKp38α appears during the early stages of mitosis, and in telophase and cytokinesis is being rapidly dephosphorylated [362]. Phosphorylation of Ser62 was found to have increased after treatment with taxol, an anti-cancer drug used in treating prostate cancer [363].

Bcl-2, a major anti-apoptotic protein, possesses a transmembrane domain (TM), which normally associates the protein with the OMM or IMM. Bcl-2 is predominantly found to be phosphorylated (at Ser70, Ser87, Thr56 and Thr74) by the MAPK kinases (p38 and ERK1/2) leading to a reduction in its anti-apoptotic activities [364,365,366]. For example, phosphorylation of Ser87 and Thr74 occur in normal human blood cells, but these two residues remain un-phosphorylated in hepatoma tumor cells, suggesting that dephosphorylation promotes Bcl-2′s anti-apoptotic activity in tumor cells [366]. It was also shown that dephosphorylated Ser87 and Thr74 seemed to be a signal for ubiquitin-dependent degradation by the 26S proteasome [367]. Bcl-2 phosphorylation at Ser70, Ser80, and Thr69 by JNK1 shown in human T lymphocytes arrested in G2/M phase in response to microtubule-damaging agents makes cells more susceptible to apoptosis [368].

Similarly, phosphorylation of the pro-survival protein Mcl-1 by JNK on residues Thr163 and Ser121 promotes apoptosis [369,370] while phosphorylation of Ser64 enhances its anti-apoptotic activity [371]; phosphorylation by ERK1/2 exhibited a similar dual effect [372]. The putative phosphorylation of the N-terminal domain of Smac/DIABLO by JNK mediates its release from the mitochondria to ubiquitinylate the inhibitors of apoptosis (IAPs) meant for degradation, thereby maintaining the apoptotic process [373,374,375]. Smac/DIABLO is also phosphorylated at Ser67 by AKT, which promotes apoptosis in HeLa cells [376]. Overall, the precise mechanism by which JNK and other kinases promote or inhibit cell survival proteins is not entirely clear and often exhibits a dual activating/inhibiting character, where phosphorylation of the same protein by one kinase may lead to opposite effects depending on the various cellular conditions (for rev. see [377].

Although Ser/Thr kinases seem to have the largest role in the Bcl-2 protein family regulated apoptosis, there is a number of other protein kinases which are also involved. The cytosolic form of LYN (cLYN) inhibits the mitochondrial apoptotic pathway, and this effect arises from its kinase activity [378,379,380]. The deactivation/inhibition of LYN by pharmacological inhibitors or siRNA in apoptosis-resistant cells causes their re-sensitization to chemotherapy-induced apoptosis [381]. Overexpressed PKC δ promotes apoptosis in neoplastic and normal keratinocytes by targeting mitochondria, triggering the release of Cytc and disturbing the membrane potential [13]. Although ABL does not contain a typical mitochondrial localization signal, protein kinase C δ (PKC δ) could bind to ABL in the endoplasmic reticulum. This PKC δ–ABL complex translocates from the ER to the mitochondria and then triggers apoptosis [382]. Cytosolic SRC, normally a proto-oncogene, possesses anti-apoptotic properties and shows increased protein levels and activity in a variety of human cancers [383]. And GSK-3β regulates mitochondria-mediated apoptosis by playing a role in determining the threshold for mitochondrial permeability transition pore opening [384].

## 9. Conclusions

Kinases and phosphatases act together in mitochondria in a precisely conducted symphony. Any disturbance of their functions by mutations or over- or under-regulation results in severe diseases such as diabetes, cancer, neurodegeneration and, consequently, apoptosis. Here we have listed those kinases that have been found to be associated with the outer mitochondrial membrane, described the effect of phosphorylation on the various components of the mitochondrial import machinery, reviewed the effect of kinases on the enzymes of the tricarboxylic acid cycle and steroid hormone biosynthesis, surveyed what is presently known of the effect of phosphorylation on each component of the oxidative phosphorylation system as well as their influence on the uncoupled respiration that occurs in brown and beige adipose tissue, examined their importance in the various systems responsible for mitochondrial protein quality control, considered their roles in mtDNA maintenance, transcription, and translation, and briefly reviewed what is known of their many roles in apoptosis.

Despite the enormous number of studies published in the last two decades describing the role of kinases in mitochondrial biogenesis, many unanswered or poorly answered questions still remain: What are the interaction partners that support or inhibit the kinases? Are mitochondrial proteins phosphorylated before or after entering the mitochondria or are both alternatives employed? How does phosphorylation or other post-translational modifications of one amino-acid residue influence the phosphorylation of another position? Can two or more kinases phosphorylate proteins simultaneously? Are certain amino acids phosphorylated more rapidly? How does the composition of the mitochondrial membrane influence kinase interactions or membrane insertion? How does phosphorylation influence the dynamics of the mitochondrial nucleoid and thus the replication, repair and transcription of mtDNA? Which kinases interact with the ribosomes and are involved in mtDNA translation? Answering these questions could substantially contribute to better understanding how mitochondria contribute to stress responses and support healthy processes in the cell as well as the background of pathological processes.

## Abbreviations

AAC	ADP/ATP carrier protein
Abf2	ARS-binding factor 2
ABL	Abelson tyrosine kinase
ACC2	acetyl-CoA carboxylase 2
AD	Alzheimer’s disease
AKAP	A-kinase anchoring protein
AKT/PKB	protein kinase B
AMPK	5′-AMP-activated protein kinase
BAT	brown adipose tissue
CAMK	Ca^2+^/calmodulin-dependent protein kinase
CDK	cyclin-dependent protein kinase
CK	casein kinase
COX	cytochrome *c* oxidase
CREB	cAMP-response element binding protein
Cytc	cytochrome *c*
DBP	dodecamer-binding protein
DRP1	dynamin-related protein 1
EF-Tu	translational elongation factor
ER	endoplasmic reticulum
ERK1/2	extracellular receptor kinase 1/2
ETC	electron transport chain
FDH	formate dehydrogenase
GSK-3β	glycogen synthase kinase 3β
HSP	heat-shock protein
IF	inhibitory factor
IMM	inner mitochondrial membrane
HIF	hypoxia-inducible transcription factor
HMG	high-mobility group
JNK	Jun N-terminal kinase
LRRK2	leucine-rich repeat kinase 2
MAPK	mitogen-activated protein kinase
MFF	mitochondrial fission factor
Mfn	mitofusin
MFP1	MAR-binding filament-like protein 1
MIA	mitochondrial intermembrane space assembly
MIEF1/2	mitochondrial elongation factor 1/2
MIM	mitochondrial import complex
MOMP	mitochondrial outer membrane permeabilization
MPP	mitochondrial processing peptidase
MPQC	mitochondrial protein quality control
mTERF1	mitochondrial termination factor 1
MTF1	mitochondrial transcription factor 1
mTOR	mammalian target of rapamycin
mtSSB	mitochondrial single-stranded DNA-binding protein
mtUPR	mitochondrial unfolded protein response
OMM	outer mitochondrial membrane
OXPHOS	oxidative phosphorylation
PAK5	p21-activated kinase 5
PARL	presenilins-associated rhomboid-like protein
PD	Parkinson’s disease
PDC	pyruvate dehydrogenase complex
PDH	pyruvate dehydrogenase
PDK	pyruvate dehydrogenase kinase
PDP	pyruvate dehydrogenase phosphatase
PGK1	phosphoglycerate kinase 1
PKA	protein kinase A
PKC	protein kinase C
PKR	protein kinase R
PINK1	*PTEN*-induced putative kinase 1
PLK3	Polo-like kinase 3
POLRMT	mitochondrial RNA polymerase
PTM	post-translational modification
ROS	reactive-oxygen species
SAM	sorting and assembly machinery
SAPK	stress-induced protein kinase
SDH	succinate dehydrogenase
SDHA	succinate dehydrogenase complex subunit A
sHSP	small heat-shock protein
StAR	steroidogenic acute regulatory protein
TCA	tricarboxylic acid cycle
TFAM	mitochondrial transcription factor A
TFB2M	mitochondrial transcription factor B
TFD	trophic factor deprivation
TIM	translocase of the inner mitochondrial membrane
TNFα	tumor necrosis factor-α
TOM	translocase of the outer mitochondrial membrane
TPKI	Tau protein kinase I
TRAP1	TNF receptor-associated protein 1
UCP1	uncoupling protein 1
VSM	vascular smooth muscle
XIAP	X-linked inhibitor-of-apoptosis protein.
